# Microstructure and adhesion characteristics of a silver nanopaste screen-printed on Si substrate

**DOI:** 10.1186/1556-276X-7-49

**Published:** 2012-01-05

**Authors:** Kwang-Seok Kim, Yongil Kim, Seung-Boo Jung

**Affiliations:** 1 SKKU Advanced Institute of Nanotechnology (SAINT), Sungkyunkwan University, 2066 Seobu-ro, Jangan-gu, Suwon-si, Gyeonggi-do, 440-746, Republic of Korea; 2 School of Advanced Materials Science and Engineering, Sungkyunkwan University, 300 Cheoncheon-dong, Jangan-gu, Suwon, 440-746, Republic of Korea

**Keywords:** silver nanopaste, screen printing, sintering, density, adhesion.

## Abstract

The microstructural evolution and the adhesion of an Ag nanopaste screen-printed on a silicon substrate were investigated as a function of sintering temperature. Through the two thermal analysis methods, such as differential scanning calorimeter and thermo-gravimetric analysis, the sintering conditions were defined where the temperature was raised from 150°C to 300°C, all with a fixed sintering time of 30 min. The microstructure and the volume of the printed Ag nanopaste were observed using a field emission scanning electron microscope and a 3-D surface profiler, respectively. The apparent density of the printed Ag nanopaste was calculated depending on the sintering conditions, and the adhesion was evaluated by a scratch test. As the sintering temperature increased from 150°C to 300°C, the apparent density and the adhesion increased by 22.7% and 43%, respectively. It is confirmed that the printed Ag nanopaste sintered at higher temperatures showed higher apparent density in the microstructural evolution and void aggregation, resulting in the lower electrical resistivity and various scratched fractures.

## Introduction

Micro and nanofabrication are essential for the modern electronic devices [1]. Recently, printed electronics has been highlighted by many researchers in academia and industry as emerging manufacturing technologies to fabricate portable and display devices [2-6]. The fabrication methods of printed electronics reported so far include direct printing techniques such as inkjet, gravure, and screen printing [7-9]. These techniques have been put forward as alternative methods for patterning conducting circuits due to the short manufacturing time, low cost, large-area patternability, and environmental friendliness compared to conventional photolithography [10]. Printed electronics is based on an additive manufacturing technology and thereby requires heat treatment after the printing process. In addition, the features of the patterns directly printed on a substrate also depend on the heat treatment. Therefore, it is essential to understand the behaviors of nanoparticles in a sintering process in order to provide an insight into the printing techniques.

Part of an ongoing research project in our laboratory is to produce printed thin films with sufficient adhesion, which is directly related to the lifetime of the electronic devices. However, it is difficult to measure the adhesion of a printed film that has a weak and thin layer, and hence, this has been one of the key issues in this project. A scratch test is the most practical method for assessing the adhesion of the thin film to the substrate [11]. This is because the critical load determined by the scratch test is widely regarded as the representative of film adhesion [12].

Based on these requirements, we investigated the effects of heat treatment on the microstructural evolution and electrical property of the screen-printed Ag nanopaste. The influence of sintering temperature on the adhesion was also characterized by the scratch test.

## Methods

**Figure 1:**
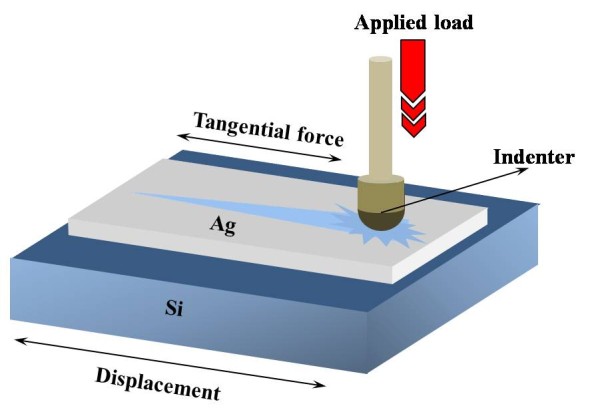
**A Schematic diagram of scratch testing**.

The Ag nanopaste (Silver nanopaste DGP, Advanced Nano Materials Inc., Kumho-ri Cheongwon-gun, South Korea) was composed of Ag nanoparticles with a mean size of 24 nm, which were dispersed in α-terpineol matrix at a solid loading of 73% by weight. The shape of the Ag nanoparticles was examined by a JEOL JEM-1200EX transmission electron microscope [TEM] (JEOL Ltd., Akishima, Tokyo, Japan). Two types of thermal analysis were performed on the Ag nanopaste: differential scanning calorimeter [DSC] and thermo-gravimetric analysis [TGA]. A screen printing machine (MT-550TV, Micro-Tec, Chiba City, Chiba, Japan) was used to duplicate the conductive patterns. The Ag nanopaste was printed using a 400-mesh screen mask onto a silicon [Si] substrate passivated with SiO_2_. All printed patterns were dried on a hot plate at 70°C for 10 min, and then sintered in a box-type, muffle furnace (RTA-BRT100, BLS Korea Inc., Seoul, South Korea) for 30 min under a sintering temperature ranging from 150°C to 300°C. A four-point probe method was adopted to measure the electrical resistivity. The apparent density of the screen-printed Ag nanopaste depending on the sintering temperatures was calculated from the precise volume and mass measured using a commercial precision scale (JL-180, Chyo Balance Corp., Minami-ku, Kyoto, Japan) and a 3-D surface profiler (Nanoview2000, Nanosystem Inc., Daejon City, South Korea), respectively. Figure 1 shows a schematic diagram of scratch testing, and the table in Additional file 1 lists the detailed scratch parameters. The scratch test was carried out using a commercial scratch tester (MSTX, CSM Instruments, Needham, MA, USA) equipped with a diamond indenter having a tip radius of 10 μm. The microstructural evolution was investigated using a field emission scanning electron microscope [FE-SEM] (JSM-7401F, JEOL Ltd., Akishima, Tokyo, Japan), and an optical microscope [OM] was used to observe the fracture surfaces of the screen-printed Ag nanopaste after the scratch test.

## Results and discussion

**Figure 2:**
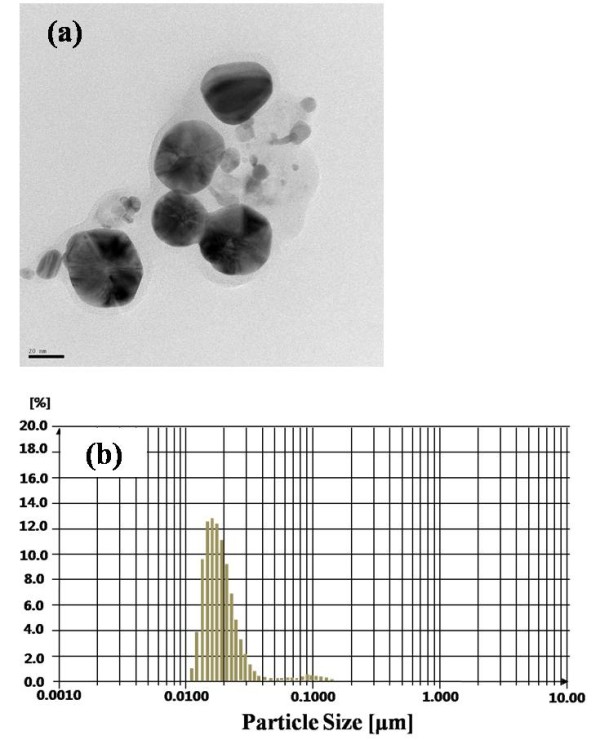
**A TEM image (a) and size distribution (b) of Ag nanoparticles**.

Figure 2a, b shows a TEM image and the measured size distribution of the Ag nanopaste, respectively. In the TEM image, most of the Ag nanoparticles have a diameter of approximately 25 nm. It was also confirmed that the diameters of the Ag nanoparticles were within 10 and 30 nm with a narrow distribution, as shown in Figure 2b. This distribution of particle size was known to be affected by three major factors, i.e., the generation rate of the Ag embryos, the growth rate of the Ag nanoparticles, and the extent of surfactant absorbing or encapsulating if no agglomeration occurred between the nanoparticles [13].

**Figure 3:**
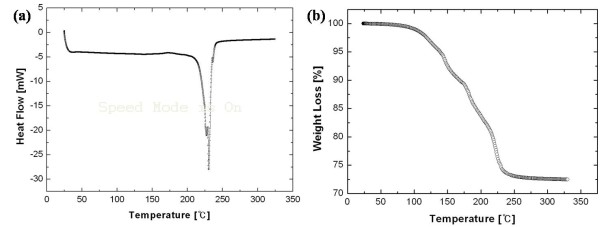
**DSC (a) and TGA (b) curves of the Ag nanopaste**.

The thermal properties of the Ag nanopaste are shown in Figure 3. The DSC curve had a slightly exothermic slope from 30°C to 200°C at the rate of 10°C/min and one sharp endothermic peak at 230°C. It means that the necking reaction in the Ag nanopaste occurred at the point of the endothermic peak, resulting in the coarsened nanoparticles. Compared with the TGA curve, any moisture and solvent in the Ag nanopaste were dried or decomposed thermally from 100°C to 300°C at the rate of 10°C/min. In this temperature range, a maximum rate of weight loss was shown at 225°C, and the total weight loss was approximately 27 wt.%. Based on these results, the sintering temperature range was determined from 150°C to 300°C to investigate the influence of heat treatment on the microstructural evolution and property variation.

**Figure 4:**
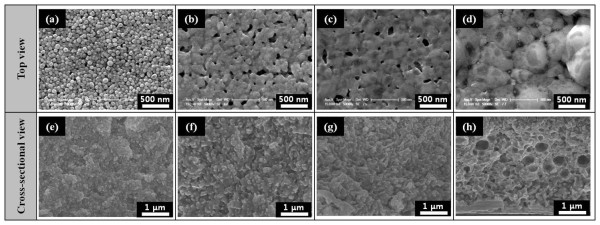
**FE-SEM micrographs of the screen-printed Ag nanopaste sintered at various temperatures**. (**a**, **e**) 150°C, (**b**, **f**) 200°C, (**c**, **g**) 250°C, and (**d**, **h**) 300°C.

The microstructural evolution as a function of sintering temperatures was shown in Figure 4. The surface of the Ag nanopaste sintered at 150°C for 30 min exhibited a similar particle shape and size compared to the as-dried one. However, when the printed Ag nanopaste was sintered at 200°C for 30 min, the clusters were built via interconnections, resulting from interparticle necking that occurred after the drying of the dispersing agent and decomposition of the organic solvent. Therefore, this microstructural evolution matched well with the thermal analysis results, as shown in Figure 3. Above a sintering temperature of 200°C, the surface of the sintered Ag nanopaste drastically changed from discrete and spherical Ag nanoparticles to continuous and consolidated ones.

**Figure 5:**
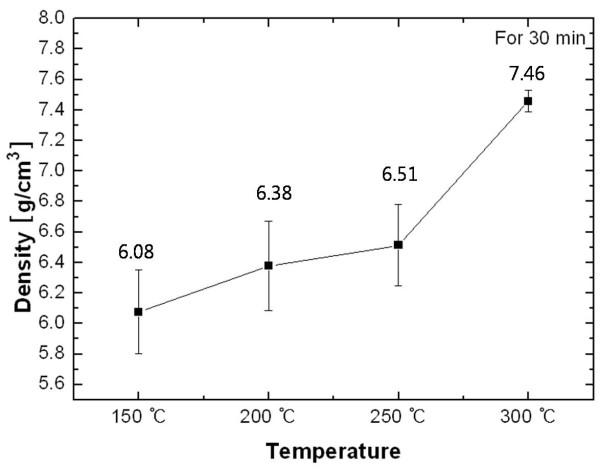
**The apparent density of the Ag nanopaste**.

Figure 5 plots the variation in the apparent density of the Ag nanopaste as a function of sintering temperature. Except at 300°C, the average apparent density did not change much which was approximately 6.32 g/cm^3 ^within the standard deviation of 0.22. At 300°C, the film of the Ag nanopaste as the result of volume shrinkage appeared after the interparticle neck growth, as shown in Figure 4d[14]. Because the apparent density was calculated from the values of the measured mass and volume, any change in mass or volume would consequently affect the density. Although some pores were observed inside the film of the Ag nanopaste, the main parameter was the volume shrinkage of the screen-printed Ag film compared to the mass decrement caused by the solvent evaporation. In this case, although the mass of the Ag nanopaste was reduced by solvent evaporation that resulted in observable pores inside the film, the dominating factor was still the volume shrinkage of the screen-printed Ag film. Therefore, the volume shrinkage led to the increase of apparent density.

**Figure 6:**
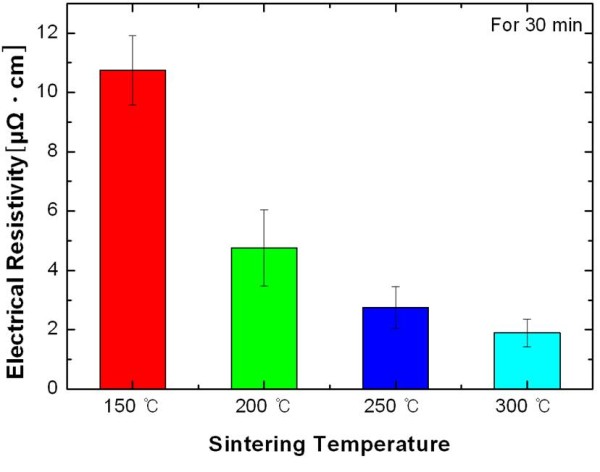
**The electrical resistivity of the Ag nanopaste**.

Figure 6 reveals the influence of the sintering temperature on the electrical resistivity of the Ag nanopaste. The electrical resistivity dramatically decreased with the increasing sintering temperature, implying a conduction pathway between the Ag nanoparticles due to the interparticle neck formation and growth. This is because the mechanism of electrical conduction in a metal nanopaste features the point-to-point contact between the conductive nanoparticles [15]. In other words, when the conductive nanoparticles have been necked to a sufficient extent, the film of the printed nanopaste becomes relatively well conductive despite of its porosity. The Ag nanopaste sintered at 300°C showed the lowest electrical resistivity of 1.89 μΩ·cm.

**Figure 7:**
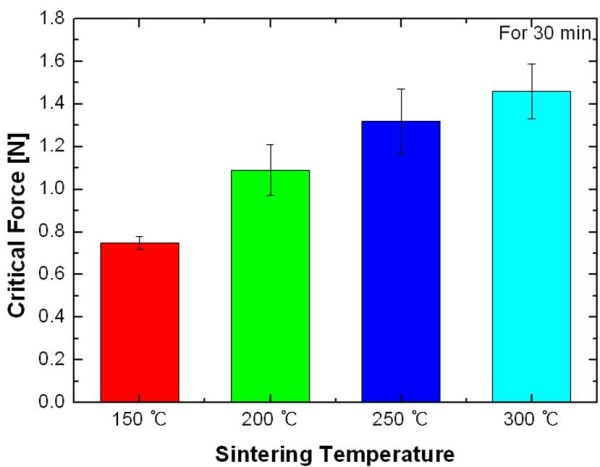
**The critical friction force of the Ag nanopaste**.

Figure 7 exhibits the adhesion strength of the Ag nanopaste printed on the Si substrate as a function of sintering temperature. A critical friction force is the force needed to pull out a film on a substrate. Overall, the critical friction force increased linearly with an increasing sintering temperature. As previously seen in Figure 4b, the nanoparticles on the surface of the Ag nanopaste formed clusters with a diameter of 130 to approximately 180 nm due to interparticle necking at a sintering temperature of 200°C. The clusters grew larger with the three-dimensional interconnections as the sintering temperature increased, which increased from around 300 nm at 250°C (Figure 4c) to 600 nm at 300°C (Figure 4d). Therefore, as the sintering temperature increased, the clusters were connected more strongly, and hence, the surface area between the Ag nanopaste and the Si substrate increased, resulting in the higher friction force.

**Figure 8:**
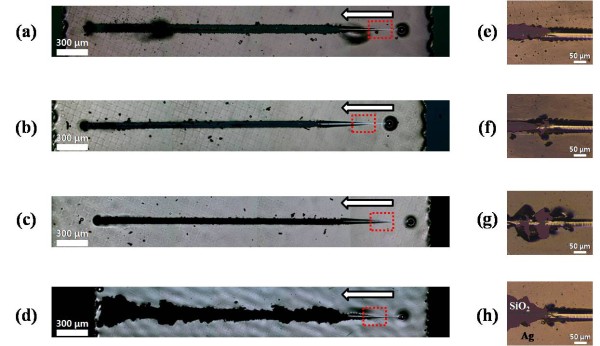
**Optical micrographs of the scratched Ag nanopaste surface sintered at various temperatures**. (**a**, **e**) 150°C, (**b**, **f**) 200°C, (**c**, **g**) 250°C, and (**d**, **h**) 300°C.

In order to investigate the scratched surface of the Ag nanopaste, the scratches were observed using the OM. The panorama images illustrate the direction of scratch testing, and the morphologies of the entire scratches are shown in Figure 8a, b, c, d. As the sintering temperature increased, larger parts of the Ag nanopaste were pulled out due to the stronger connections in the Ag clusters. Figure 8e, f, g, h indicates the exact starting points to pull the film out from the substrate. The different fracture modes were identified depending on the sintering temperature. The printed Ag nanopaste sintered at 300°C exhibited a fracture behavior like a bulk Ag film.

## Conclusions

The characteristics of thin printed patterns are dominated by the heat treatment applied. The influence of sintering temperature on the adhesion of the screen-printed Ag nanopaste was investigated. The scratch test, which is to measure the critical friction force of the film, was suggested to be a suitable method to evaluate the adhesion of printed patterns. Overall, the critical friction force increased by 43% as the sintering temperature increased from 150°C to 300°C. To rationalize these experimental results, the microstructural evolution and variation of density were investigated as a function of sintering temperature. The Ag nanopaste sintered at higher temperatures showed the accelerated condition. The calculated apparent density of the Ag nanopaste increased from 6.08 g/cm^3 ^at 150°C to 7.46 g/cm^3 ^at 300°C. It was concluded that the printed Ag films sintered at higher temperatures became more densely packed, which resulted in the lower electrical resistivity and the stronger adhesion of the printed Ag nanopaste.

## Abbreviations

DSC: differential scanning calorimeter; FE-SEM: field emission scanning electron microscope; OM: optical microscope; TEM: transmission electron microscope; TGA: thermo-gravimetric analysis

## Competing interests

The authors declare that they have no competing interests.

## Authors' contributions

KSK carried out the density measurement and scratch test and wrote the manuscript. YK carried out the two thermal analysis of the Ag nanopaste and participated in the screen printing. SBJ participated in the design and coordination of this research. All authors read and approved the final manuscript.

## Supplementary Material

Additional file 1 **The parameters of scratch test**. A table listing the detailed scratch parameters. Click here for file
