# Supplementary figures and images for: Microstructure and adhesion characteristics of a silver nanopaste screen-printed on Si substrate
Source: Nanoscale Res Lett. 2012 Jan 5;7(1):49. doi: 10.1186/1556-276X-7-49 (PMC3265413; doi:10.1186/1556-276X-7-49)

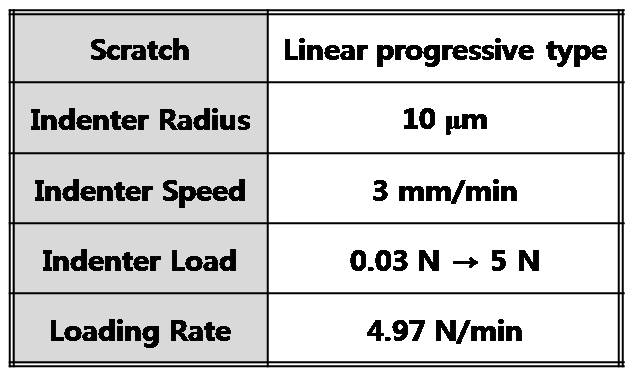

Supplement: Additional file 1 — The parameters of scratch test. A table listing the detailed scratch parameters. [file 1556-276X-7-49-S1.JPEG]
